# Outcome of Open Reduction Alone or with Concomitant Bony Procedures for Developmental Dysplasia of the Hip (DDH)

**DOI:** 10.3390/children9081213

**Published:** 2022-08-12

**Authors:** Kamal Jamil, Rostam Saharuddin, Ahmad Fazly Abd Rasid, Abdul Halim Abd Rashid, Sharaf Ibrahim

**Affiliations:** Department of Orthopaedics & Traumatology, Faculty of Medicine, Universiti Kebangsaan Malaysia, Cheras, Kuala Lumpur 56000, Malaysia

**Keywords:** developmental dysplasia of the hip, open reduction, pelvic osteotomy, femoral osteotomy, hip dysplasia

## Abstract

Introduction: Developmental dysplasia of the hip (DDH) is commonly managed in a tertiary centre and regularly involves surgical treatment. The aim of this study is to determine the surgical outcome of DDH patient treated with either open reduction alone or combined with bony procedures in our institution. Methods: Medical records of DDH patients treated surgically were reviewed. Patients were divided into two groups: Group A: underwent open reduction (OR) only; and Group B: underwent open reduction with additional bony procedures (ORB), such as pelvic or femoral osteotomy. Modified McKay classification was used to evaluate the clinical outcome, and Severin classification for the radiological outcome. Presence of avascular necrosis and other post-operative complications were recorded. Results: A total of 66 patients (76 hips) were reviewed with the mean age of 11.9 ± 4.8 years. Mean duration of follow up was 8.6 ± 4.7 years (ranged 2 to 23 years). From our sample, 50/66 patients (75.8%) achieved satisfactory clinical outcome, whereas 48/66 patients (72.7%) had satisfactory radiological outcome. A higher proportion of patients achieved satisfactory outcomes in the OR group compared to the ORB group (*p* < 0.05), but no difference was seen in terms of radiological outcome (*p* = 0.80). Overall, 23 hips (34.8%) developed radiographic evidence of avascular necrosis (AVN). Nineteen hips had undergone ORB, although they were mainly (63.2%) Grade I AVN. Incidence of AVN was comparable in both groups (*p* = 0.63), but presence of AVN led to a higher proportion of unsatisfactory clinical and radiological outcome (*p* < 0.05). Other complications included redislocation/subluxation (13.6%) and bleeding (0.1%). Conclusions: Good overall outcome of DDH surgery was achieved in our centre. The OR group may produce a better clinical outcome, but with similar radiological results and AVN rate with the ORB group. The presence of AVN is associated with unsatisfactory clinical and radiological outcomes.

## 1. Introduction

The incidence of DDH varies among different regions and population. In Malaysia, the incidence of DDH was reported at 0.7 cases per 1000 births [1]. Other countries which implement universal ultrasonographic screening of DDH reported increasing cases, up to 25 to 50 cases per 1000 birth [2]. Once diagnosed, the aim of the DDH treatment is to provide a concentric and stable hip [2,3,4]. Early initiation of treatment is essential to allow optimal hip remodelling. If left untreated, the dysplastic hip may lead to dislocation and the child will have an abnormal gait resulting in hip pain. In the long term, early onset of osteoarthritis is expected in these cases [5].

Management of DDH is mainly based on the age of presentation. Pavlik harness can be initiated during the neonatal period. Closed reduction and hip spica are performed between the ages of 6 to 12 months old if the dislocation persisted. Open reduction with or without pelvic and femur osteotomy is reserved for cases of failed closed reduction and for patients with late presentations [3,6]. Successful management of DDH can be reviewed based on the radiographs and clinical assessments.

Treatment by either closed or open reduction has been shown to lead to favourable outcomes in many short- to middle-term studies [7,8,9]. In a walking-age child with DDH, open reduction with concomitant pelvic osteotomy would provide the best results according to a recent systematic review [10]. Long-term studies suggested that older age at first surgery and complication of avascular necrosis are associated with poor outcomes [5,11]. However, there are not many long-term studies on DDH in Malaysia and to the best of our knowledge there is only one study on the treatment outcome [12]. Therefore, we decided to review the clinical and radiological outcome of the DDH patients that were surgically treated at our centre for the past 20 years. We also want to determine whether an open reduction alone would lead to a similar outcome to a combined surgery with bony procedures.

## 2. Materials and Methods

**Figure 1:**
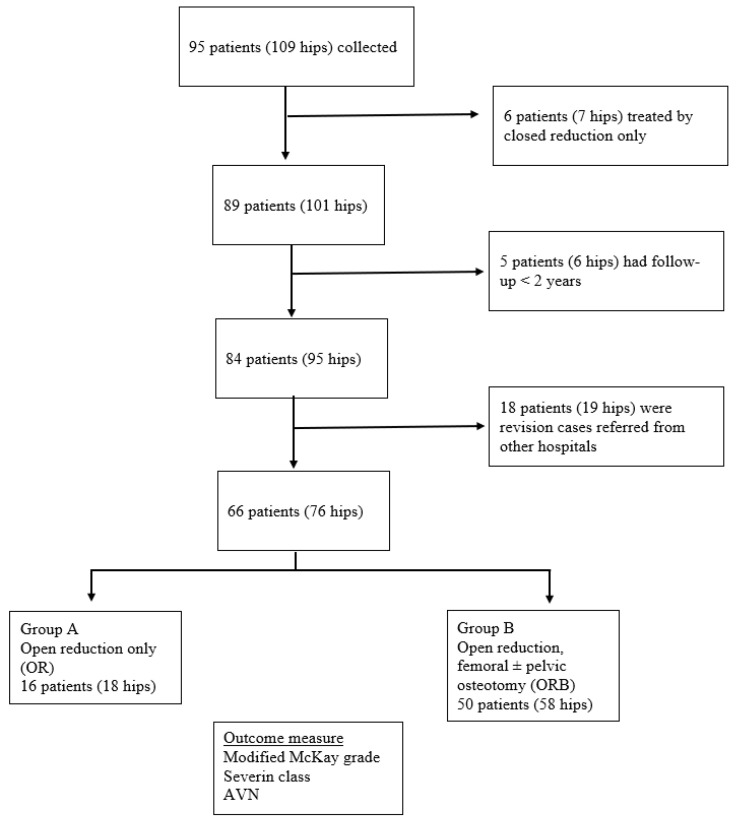
Flowchart of the patient selection and study design.

Following the approval of the institutional ethics committee board, we retrospectively reviewed the medical records of DDH patients who were surgically treated in our centre since January 1996 to January 2018. All bilateral or unilateral DDH patients operated on during the study period were included in this study. Patients with teratological, paralytic, septic, or traumatic hip dislocations were excluded. All patients included had at least two years of follow-up from the operation date. Figure 1 shows the flowchart of our patient selection and study design.

Patients were divided based on the surgical treatment received. Group A consists of patients who underwent open reduction (OR) only, whilst patients in Group B underwent open reduction with either femoral or pelvic osteotomy (ORB). All patients underwent open reduction with the modified Smith-Petersen approach (anterior approach with bikini skin incision). In cases where there was significant tension of the surrounding soft tissue impeding reduction, femoral shortening was performed following the technique described by Klisic [13]. Pelvic osteotomies (either Salter [14] or Pemberton [15]) were carried out for patients who had poor femoral head coverage following hip reduction. For both groups of patients, the child was immobilized in hip spica for a total duration of 3 months post-operatively. We did not practice pre-operative traction. Surgery was performed by two senior paediatric orthopaedic surgeons (SI and AHR).

The outcome measures were assessed by reviewing the documented clinical data and follow-up radiographs. Modified McKay [16] grading was used to evaluate the clinical outcome of each group. We consider McKay grade I and II (‘excellent’ and ‘good’) as ‘satisfactory’, whereas grade III and IV (‘fair and poor’) are considered as ‘unsatisfactory’ outcomes. The Severin [17] classification was used to evaluate the radiological outcome, whereby class I and II were considered ‘satisfactory’ and class III and IV were ‘unsatisfactory’. Intra-operative and post-operative complications were reviewed from the medical records. Any evidence of AVN of the hip was classified using Bucholz and Ogden [18] classification. Data collection was performed by a single researcher. For the purpose of analysis, data on the ‘worst’ hip were taken for cases with bilateral DDH.

The data collected were analyzed using the SPSS software version 21.0. The chi-square and Fishers’ exact test were used for categorical data. The *p*-value of < 0.05 was considered as statistically significant.

## 3. Results

A total of 66 patients (76 hips) were selected with the mean age at surgery of 3.8 ± 2.6 years and 11.9 ± 4.8 years at the last follow-up. The mean duration of follow up was 8.6 ± 4.7 years (ranged 2 to 23 years). The majority of patients were females; 57 patients (86.4%) and there were 9 males (13.6%). Table 1 shows the demographic characteristics of the subjects. Group B consisted of children who were significantly older than Group A.

At the latest follow-up visit, the clinical outcome by modified McKay grading revealed that 46 patients (69.7%) were grade I (excellent), four (6.1%) were grade II (good) and 16 (24.2%) were grade III (fair). None of the patients were graded as poor, which is grade IV.

**Figure 2:**
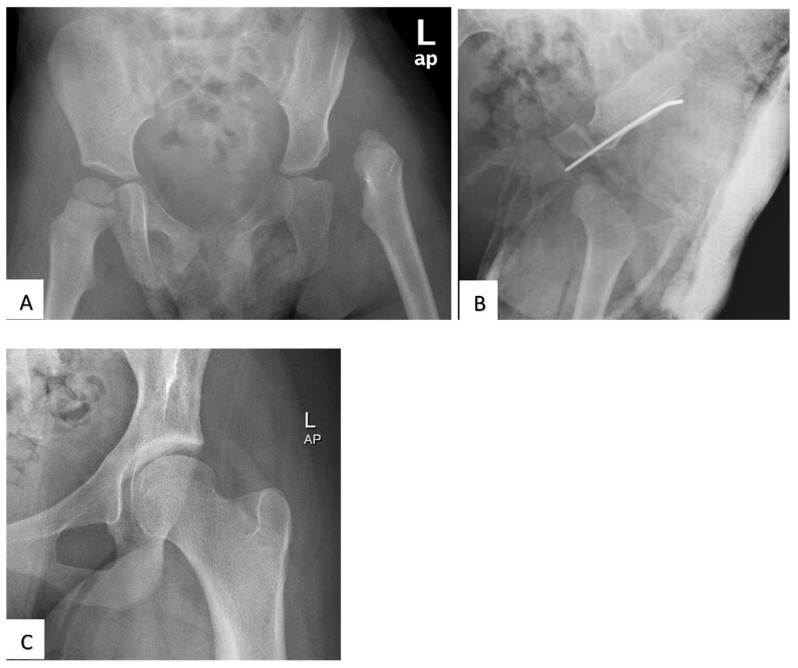
Radiographs of a boy treated with open reduction and Salter osteotomy. He had an excellent clinical outcome (McKay grading). (**A**) Pre-operative anteroposterior pelvic radiograph at age 20 months showing a left hip dislocation and a dysplastic left acetabulum. (**B**) Post-operative hip radiograph shows hip reduction and the Salter osteotomy stabilised with a K-wire. (**C**) Anteroposterior left hip radiograph showing a normal femoral head shape and acetabulum (Severin class I) at age 11 years old.

**Figure 3:**
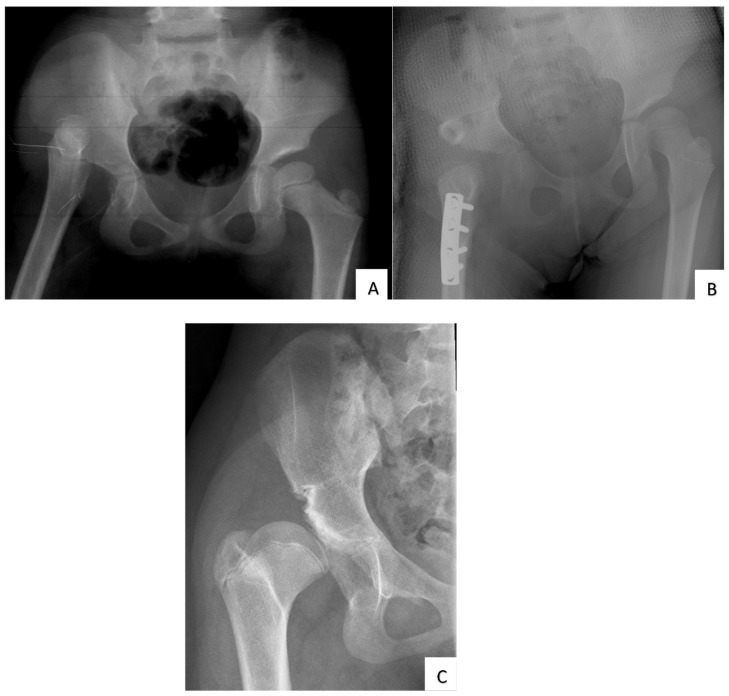
Radiographs of a girl treated with open reduction, femoral shortening, and Salter osteotomy. (**A**) Pre-operative pelvic radiograph at age 6 years old showing a high right hip dislocation and dysplastic acetabulum. (**B**) Post-operative radiograph shows the hip is reduced but retroverted. Salter osteotomy with a bone graft taken from the resected femoral shaft. The K-wire fixing the bone graft has been removed. (**C**) At the age of 13 years, the radiographic outcome is poor (Severin class IV). There are presence of coxa breva, femoral head subluxation, and severe acetabular dysplasia. Her clinical outcome by McKay grading was also poor.

The overall radiological outcome showed that 41 patients (62.1%) had Severin class I, seven patients (10.6%) class II, two (3%) class III and 16 (24.2%) class IV. In total, 23 hips (34.8%) developed radiographic evidence of AVN as classified by Bucholz and Ogden. The majority were grade I (21.2%), followed by grade II (10.6%), and grade III (3%). Figure 2A–C shows a case example of a patient with a satisfactory outcome, while Figure 3 is an example of an unsatisfactory outcome.

Chi-square test found that the rate of unsatisfactory outcome was significantly lower in the OR group than the ORB (*p* < 0.05) for the clinical outcome (Table 2). However, both groups showed a comparable radiological result by Severin class (*p* = 0.80). There was no difference in the incidence of AVN between both groups (χ^2^ =2.66, *p* = 0.63). Although, presence of AVN led to a higher proportion of unsatisfactory clinical and radiological outcome (*p* < 0.05) (Table 3).

We also investigated whether age at the time of primary surgery would influence the outcome. Comparison between patients operated upon before and after the age of 3 years old revealed that there were no differences in terms of McKay grading (*p* = 0.34), Severin class (*p* = 0.23), and also incidence of AVN (*p* = 0.28).

There were seven patients (eight hips) who had had multiple procedures before the latest follow-up. These patients either had repeat open reduction (one hip) or repeat open reduction with additional femoral/pelvic procedure (seven hips). They also represent the hips which had subluxated (five hips) or redislocated (three hips) in our cohort. Apart from that, nine other patients (11 hips) had history of prior closed reduction (but redislocated) before the index procedure. However, we found that the patients who had multiple procedures showed a comparable outcome with those patients who underwent a single procedure only, in terms of McKay grading (*p* = 0.29), Severin class (*p* = 0.38), and also incidence of AVN (*p* = 0.14). Other complication includes intra-operative blood loss requiring blood transfusion (four hips).

## 4. Discussion

Our results indicate that the majority of the patients achieved satisfactory clinical (75.8%) and radiological (72.7%) outcome in the medium to long-term, following the surgical treatment with open reduction with or without femoral/pelvic procedure. We construe ‘satisfactory’ outcome as ‘excellent’ or ‘good’ in McKay clinical grading and Severin I and II class for the radiological outcome. Other studies reported their satisfactory outcomes in the range of 70–90%, with various DDH procedures performed and different duration of follow-ups [7,8,19,20,21,22,23,24,25]. Many of the authors presented results of a single-stage procedure, combining open reduction with femoral and pelvic osteotomies [7,19,20,21,22,23]. Medium-term studies showed high percentages of satisfactory outcomes (80–90%) for a single-stage procedure, with average duration of follow-up of 3 to 6 years [7,19,24,25,26]. In long term studies, few researchers have shown comparable results. Varner et al. reviewed 57 patients at skeletal maturity, with a mean duration of 18 years follow-up and found 78% satisfactory radiological outcome [8]. Similarly, Wu et al. achieved about 80% satisfactory clinical and radiological outcome in children treated with open reduction and Pemberton osteotomy at an 11-years-average follow-up [11].

In our study, we found that children who underwent open reduction alone (OR) fared better clinically compared to children who had additional bony procedures (ORB). This is in line with the findings of a recent systematic review which reported that the odds of having a satisfactory clinical and radiological outcome is higher in the OR group [10]. Though, we did not find any difference in the radiological outcome between the groups in our study. The same systematic review also suggested that even though the OR group has a good outcome, they presented with an unacceptably high rates of reoperation, whereby the odds of needing further non-salvage procedures are as high as 10–15 times compared to ORB [10]. We have a relatively small number of patients in the OR group (16 patients), but all of them did not require additional surgeries.

One of the reasons why the OR group might perform better than the ORB is due to the younger age at surgery. It is generally accepted that ORB procedures are indicated for children of at least 18 months old, therefore the age differences compared to the OR group is unavoidable. In a Turkish study, children in the OR group aged below 18 months old had less AVN and reoperation rates in comparison to the ORB group who were older than 18 months [9]. However, there was no differences in their clinical and radiological outcome. Age was also found as an independent risk factor for unsatisfactory hip function in other studies [24,25,27,28,29]. Older age children have less potential of acetabular remodelling and may require more complex surgery for their treatment. Holman et al. reviewed 57 patients at an average age of 30 years old and found that radiological results at maturity deteriorates in children who were operated after the age of 3 years [5]. We are unable to show this age effect in our study but many of our patients have not reach skeletal maturity at the latest follow-up. Ning et al. further divided their patients into three age groups: 1.5 to 2.5 years, 2.5 to 8 years and above 8 years old during the index surgery [25]. Evaluating a large group of over 800 hips, they found that a single stage procedure at the age between 2.5 to 8 years produced the best radiological outcome with low AVN rates.

AVN is the most serious complication from DDH surgery. Post-operative AVN has been reported to be as low as 0% but up to 67% in various studies [30]. This discrepancy is attributable to the various classifications used to describe this complication. Kalamchi and McEwen [31] and Bucholz and Ogden [18] are the two popular classifications for AVN whereby type I class in both classifications would represent temporary epiphyseal mottling or fragmentation. Type II and above is when the physeal damage is apparent and therefore could possibly lead to growth disturbances and premature arthritis as young adults. Our data show clinically relevant AVN in 13.6% of the cases. We found that the incidence was comparable between the OR and ORB groups, although the presence of AVN was associated with unsatisfactory clinical and radiological outcomes. Our results are in line with the recent systematic review which also failed to find a statistical difference between OR alone and OR with either femoral or pelvic osteotomy in terms of AVN incidence [10]. However, if a single-stage procedure was performed instead (which involved OR with both femoral and pelvic procedures), significantly higher rates of AVN were seen as compared to OR alone. Other studies also associate AVN with unfavourable post-operative outcomes [5,11,24,25,30].

The reason for developing post-operative AVN is still debatable but there are some postulated risk factors. Older age at surgery, higher grade of dislocation, surgical approach (anterior vs medial), performing pelvic osteotomy, and extreme hip position in spica have all been proposed to increase the risk of AVN [26]. Wu et al. investigated the presence of AVN in 167 hips treated with OR and Pemberton osteotomy [11]. They proposed that excessive reduction leading to inferior displacement of the femoral head may compress the lateral epiphyseal artery, thus causing AVN. There is a general belief that performing a femoral shortening can effectively reduce soft tissue tension therefore able to avoid AVN in DDH surgery, although this has not been proven to be true [10]. Despite that, the procedure may be needed in older children with proximal femoral migration to allow concentric hip reduction. Apart from that, multiple surgeries theoretically would disrupt the blood supply to the hip and lead to AVN. However, we were unable to show that repeated procedures are related to the incidence of AVN in our cohort.

The other challenging complication encountered post operatively is redislocation of the hip. We had 7/66 patients or 10.6% of cases that needed to be re-operated for this reason. Other studies reported their redislocation rates around 0–8% [25,32]. Failure to get the hip reduced deep into the acetabulum is the main reason for redislocation [26]. This may be due to severe joint laxity or increased femoral anteversion. If hip is unstable following reduction and capsulorrhaphy, additional measures such as femoral or pelvic osteotomy might be needed to ensure deep positioning of the femoral head at the primary surgery. Ganger et al. proposed meticulous analysis with CT scan or MRI to assess degree of femoral anteversion, labral abnormality, and acetabular version in redislocated cases [26]. Revision surgery for redislocated hip is technically demanding and should be performed by an experienced surgeon.

The results of this study are based on patients operated upon by two senior orthopaedic surgeons who might have differences in surgical technique but followed a similar treatment algorithm. However, our study is limited by its retrospective design and relatively small number of subjects in the OR group. Measurements on radiographs were performed by a single researcher, but we did not perform reliability study prior to data collection. We also excluded patients who had been operated elsewhere and referred to us, whose results might influence the outcome of this study. We had difficulty in comparing our results with other studies due to the different age groups, duration of follow-up, surgical procedure, and techniques. This issue was also reported by other authors [10,19]. However, our results of 70% satisfactory clinical and radiological outcome with a 13.6% AVN rate is in a broad agreement with other studies [9,22,23,26]. We only included patients with at least 2 years of follow-up, as that is the minimum duration to assess AVN [9]. Although, Domzalski and Synder suggested that vascular changes may not be evident until as late as 11 years after the reduction therefore follow-up less than 10 years may not reflect the true prevalence of AVN [30]. We did not identify the cause of AVN in our study. To reduce the risk of AVN, we believe that the surgical principle in DDH which is ‘concentric reduction without tension’ should be strictly adhered to, even if additional bony procedures may need to be performed to achieve it. In addition, meticulous surgical technique by highly skilled surgeon is paramount in difficult cases.

## 5. Conclusions

The outcome of DDH surgery in our centre is comparable to other studies. The OR group may produce a better clinical outcome, but with similar radiological consequence and AVN rate with the ORB group.

## Figures and Tables

**Table 1:** Demography of the study subjects. OR = open reduction, ORB = open reduction, femoral procedure ± pelvic osteotomy.

	OR (*n* = 16)	ORB (*n* = 50)	*p*-Value
Age at surgery in years			
(mean ± SD)	1.5 ± 0.4	4.5 ± 2.5	<0.05
Age at final follow-up (mean ± SD)	8.6 ± 4.6	13.0 ± 4.4	<0.05
Gender: N (%)			
Male	1 (1.5%)	15 (22.7%)	0.43 *
Female	21 (41.2%)	42 (63.6%)	
Laterality: N (%)			
Left	7 (10.6%)	28 (10.6%)	0.50 *
Right	7 (56.9%)	14 (21.2%)	
Bilateral	2 (3.0 %)	8 (12.1%)	

* Fisher exact test.

**Table 2:** Comparison between Group A (OR) and Group B (ORB) according to modified McKay grading and Severin classification. McKay grade I and II (excellent and good) = satisfactory, grade III and IV (fair and poor) = unsatisfactory outcome. The Severin classification class I and II = satisfactory and class III and IV = unsatisfactory. OR = open reduction; ORB = open reduction + femoral procedure with or without pelvic osteotomy.

Group	McKay Grading		Total	χ^2^	*p*-Value
	Satisfactory	Unsatisfactory			
OR	15	1	16		
	(22.7%)	(1.5%)	(24.2%)		
ORB	33	17	50	4.71	<0.05 *
	(50.0%	(25.8%)	(75.8%)		
Total	48	18	66		
	(72.7%)	(27.3%)			
	Severin Class				
	Satisfactory	Unsatisfactory			
OR	11	5	16		
	(16.7%)	(7.6%)	(24.2%)		
ORB	36	14	50	0.06	0.80
	(54.5%)	(21.2%)	(75.8%)		
Total	47	19	66		
	(71.2%)	(28.8%)			

* Fisher exact test.

**Table 3:** Correlation between presence of AVN and outcome according to modified McKay grading and Severin classification. McKay grade I and II (excellent and good) = satisfactory, grade III and IV (fair and poor) = unsatisfactory outcome. The Severin classification class I and II = satisfactory and class III and IV = unsatisfactory. AVN = avascular necrosis.

AVN	McKay Grading		Total	χ^2^	*p*-Value
	Satisfactory	Unsatisfactory			
No AVN	36	8	43		
	(53.0%)	(12.1%)	(65.2%)		
Grade 1	9	5	14	8.32	<0.05 *
	(13.6%)	(7.6%)	(21.2%)		
Grade 2	4 (6.1%)	3 (4.5%)	7 (10.6%)		
Grade 3	0	2 (3.0%)	2 (3.0%)		
Total	48	18	66		
	(72.7%)	(27.3%)			
	Severin Class				
	Satisfactory	Unsatisfactory			
No AVN	36	7	43		
	(54.5%)	(10.6%)	(65.2%)		
Grade 1	6	8	14		
	(9.1%)	(12.1%)	(21.2%)	13.72	<0.05 *
Grade 2	5	2	7		
	(7.6%)	(3.0%)	(10.6%)		
Grade 3	0	2	2		
		(3.0%)	(3.0%)		
Total	47	19	66		
	(71.2%)	(28.8%)			

* Fisher exact test.

## Data Availability

The data presented in this study are available on request from the corresponding author. The data are not publicly available because they contain potentially identifying or sensitive patient information.
